# Evaluation of peripheral bronchiole visualization using model-based iterative reconstruction in quarter-detector computed tomography

**DOI:** 10.1371/journal.pone.0239459

**Published:** 2020-09-18

**Authors:** Yukiko Usui, Ryo Kurokawa, Eriko Maeda, Harushi Mori, Shiori Amemiya, Jiro Sato, Kenji Ino, Rumiko Torigoe, Osamu Abe

**Affiliations:** 1 Department of Radiology, Graduate School of Medicine, The University of Tokyo, Tokyo, Japan; 2 Department of Radiation Technology, The University of Tokyo Hospital, Tokyo, Japan; 3 Canon Medical Systems Corporation, Otawara, Tochigi Prefecture, Japan; Chongqing University, CHINA

## Abstract

This study aimed to evaluate the visualization of peripheral bronchioles in normal lungs via quarter-detector computed tomography (QDCT). Visualization of bronchioles within 10 mm from the pleura is considered a sign of bronchiectasis. However, it is not known peripheral bronchioles how close to the pleura in normal lungs can be tracked using QDCT. This study included 228 parts in 76 lungs from 38 consecutive patients who underwent QDCT. Reconstruction was performed with different thicknesses, increments, and matrix sizes: 0.5-mm thickness and increment with 512 and 1024 matrixes (Group5 and Group10, respectively) and 0.25-mm thickness and increment with 1024 matrix (Group10Thin). The distance between the most peripheral bronchiole visible and the pleura was determined in the three groups. The distance between the peripheral bronchial duct ends and the nearest pleural surface were significantly shorter in the order of Group10Thin, Group10, and Group5, and the mean distances from the pleura in Group10Thin and Group10 were shorter than 10 mm. These findings suggest the visualization of peripheral bronchioles in QDCT was better with a 1024 axial matrix than with a 512 matrix, and with a 0.25-mm slice thickness/increment than with a 0.5-mm slice thickness/increment. Our study also indicates bronchioles within 10 mm of the pleura do not necessarily indicate pathology.

## Introduction

Quarter-detector computed tomography (QDCT) has a detector size of 0.25 × 0.25 mm, which is quarter the size of a conventional multidetector-row CT, and has two-fold greater spatial resolution in the in-plane/body-axis direction. Therefore, it is possible to obtain detailed biometric information that cannot be detected using conventional CT using QDCT. Previous studies have reported that QDCT provides high spatial resolution in many regions of the human body, such as temporal bones, intracranial arteries, coronary arteries, and wrists [1–4].

For the lungs, few studies have reported image quality improvement using QDCT. Yanagawa et al. have reported that spatial resolution is better and information regarding lung anatomy is more detailed using QDCT than using conventional CT [5]. Additionally, Kakinuma et al. have reported that image quality is better using QDCT than using conventional CT for the margins of subsolid and solid lung nodules, edges of solid components and pulmonary vessels in subsolid lung nodules, air bronchograms, pleural indentations, margins of pulmonary vessels, edges of bronchi, and interlobar fissures [6]. Moreover, Tanabe et al. have reported that QDCT allows the evaluation of peripheral bronchioles that are 1–2 mm in diameter [7]. However, in normal lungs, it is not known how close to the pleura peripheral bronchioles can be tracked using QDCT.

Conventional CT techniques do not generally allow the visualization of peripheral bronchioles within 10 mm from the pleura in normal lungs. Kang et al. have reported that 21 of 41 lung lobes with pathological bronchiectasis had visible peripheral bronchioles within 10 mm from the pleura on single-slice CT [8]. The visualization of bronchioles within 10 mm from the pleura is considered a sign of bronchiectasis and is used as one of the criteria for the diagnosis of bronchiectasis according to the British Thoracic Society Guidelines for Bronchiectasis in Adults [9, 10]. Despite the continued improvement in CT performance since these reports, no new standards for the visualization of peripheral bronchioles have been proposed.

The purpose of this study was to evaluate the visualization of peripheral bronchioles in normal lungs using QDCT. We compared the visualization ability using different axial matrix sizes, as well as different thicknesses and increments in QDCT. We also investigated whether peripheral bronchioles are detected within 10 mm from the pleura, which has been considered to be a pathologic finding.

## Materials and methods

This retrospective study was approved by the Graduate School of Medicine and Faculty of Medicine, The University of Tokyo Research Ethics Committee [IRB 2561-(16)], and the requirement for informed consent from the study participants was waived because of the retrospective nature.

### Patients

During January 2018 and June 2020, 96 consecutive adult patients underwent lung CT in the University of Tokyo Hospital. Among these patients, 58 were excluded because of not having scanned in the super high-resolution mode (n = 31), emphysema (n = 11), interstitial pneumonia (n = 6), large amount of pleural effusion (n = 4), widespread pneumonia (n = 2), post lobectomy (n = 2), and not having scanned in the dorsal position (n = 2). Finally, 38 patients with 228 parts (3 parts per lung) in 76 lungs were included in the analysis.

The indications for CT were follow-up of lung nodules(n = 19), screening or follow-up examination of lung metastasis (n = 9), investigation of fever(n = 6), follow-up after mediastinal tumor surgery (n = 1), investigation of a mediastinal mass (n = 1), abnormal shadow on chest radiography (n = 1), and lung screening for collagen disease (n = 1).

### CT data acquisition

All patients underwent QDCT (Aquilion Precision; Canon, Tochigi, Japan) in the super high-resolution mode, in which 1792 detector channels are used and 512 × 512, 1024 × 1024, and 2048 × 2048 matrix sizes and 0.25-mm slice thickness are available. The acquisition parameters were as follows: detector configuration, 160 × 0.25 mm; tube potential, 120 kV; tube current–time product determined by auto exposure control. For all CT scans, full iterative reconstruction (Forward projected model-based Iterative Reconstruction SoluTion; FIRST) in the LUNG mild mode was used.

The slice thickness and slice increment were both 0.5 mm, and the axial matrix was 512 × 512 (Group5) or 1024 × 1024 (Group10). In Group10Thin, the slice thickness and slice increment were both 0.25 mm and the axial matrix was 1024 × 1024. The matrix size 1024 × 1024 is the smallest matrix available for FIRST. The region of interest was set to 225 mm to cover the entire unilateral lung. The effective radiation dose was derived by multiplying the dose length product by the chest conversion coefficient (k = 0.014 mSv/mGy/cm) [11].

### Image analysis

Image assessment was performed by two thoracic radiologists (6 and 17 years of experience). A monitor with a screen resolution of 1200 × 1600 was adopted for evaluation, using CENTRICITY Radiology RA1000 (GE Healthcare, Milwaukee, WI, USA).

**Fig 1:**
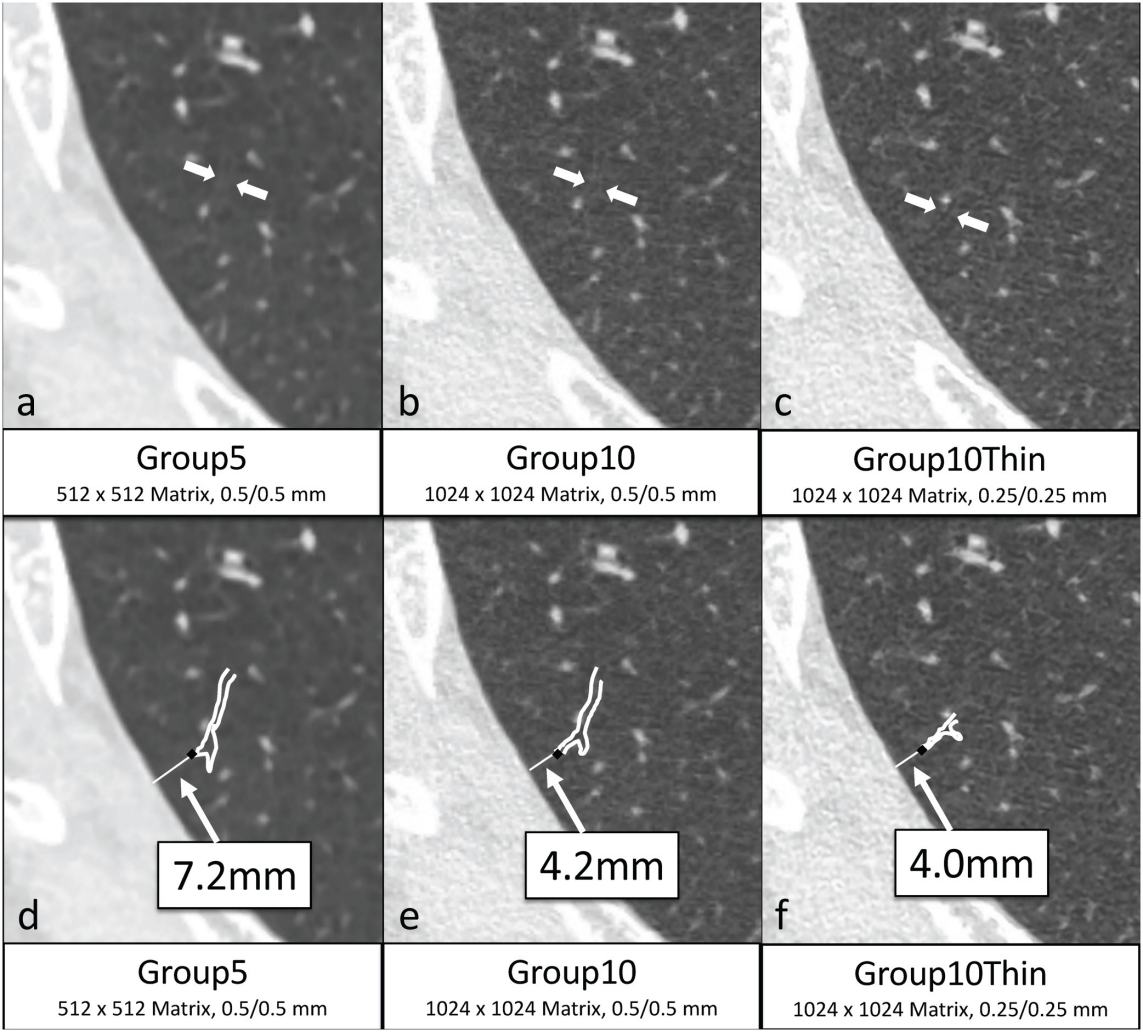
Images of lung fields are reconstructed with different thicknesses, increments, and matrix sizes, which are named as Group5 (a), Group10 (b), and Group10Thin (c). First, we chose a bronchiole randomly in Group5(a) (short arrow), and tracked the duct to the most peripherally visible end in Group5 (d) (black dot). Then, tracked the same bronchiole to its end in Group10(b) and Group10Thin(c) (short arrows). The distances between the bronchiole ends and the pleura in Group5(d), Group10(e), and Group10Thin(f) were measured (7.2 mm, 4.2 mm, and 4.0 mm, respectively in this case, long arrows). Image (d), (e), and (f) are the same slice images of image (a), (b), and (c), respectively, with annotations. Thick white lines and black dots show visible bronchiole ducts and their ends, respectively. Thin white lines show the distances between the ends and the pleura. The numbers below images mean matrix size and slice thickness / increment (mm).

Initially, a bronchiole running transversely close to the pleura was randomly selected by the two readers in agreement from Group5. Bronchioles were selected in the upper, middle, and lower lung fields of both lungs in every patient. Then, the readers identified the same bronchioles in Group10 and Group10Thin. Thereafter, the readers tracked the most peripheral bronchiole ends in the three groups. The bronchiole and bronchial lumen were defined as the two parallel bronchial wall lines identified and the low-density area between them, respectively. The point at which the visual bronchial lumen could not be followed was considered the bronchial end in this study. The distance between the visible tube end and the nearest pleural surface was measured by consensus among the readers and was recorded (Fig 1). The number of bronchial ends within 10 mm of the pleura was counted.

### Statistical analysis

The distances between peripheral bronchial ends and the nearest pleural surface in Group5, Group10, and Group10Thin was compared using Friedman test and post-hoc Wilcoxon signed-rank test while correcting for multiple comparisons using Bonferroni’s method. All *p*-values correspond to two-sided tests and the statistical significance level was set at family wise error-corrected *p* <0.05. Statistical analyses were performed using JMP Pro (version 15.0.0; SAS, Cary, NC, USA).

## Results

The patient profiles are summarized in Table 1. The distance between the peripheral bronchial duct ends and the nearest pleural surface were significantly shorter in the order of Group10Thin, Group10, and Group5 (mm, mean ± standard deviation: 8.5 ± 0.05, 8.9 ± 0.04, and 10.5 ± 0.07, respectively; p < 0.0001 for each pair).

**Table 1:** Summary of patient characteristics.

Age (year, mean ± standard deviation)	65.6 ± 13.6 (36–86)
Sex (male/female)	23/15
The reason for CT (%)	
follow-up of lung nodules	19/38 (50.0%)
screening or follow-up examination of lung metastasis	9/38 (23.7%)
investigation of fever	6/38 (15.8%)
follow-up examination after mediastinal surgery	1/38 (2.6%)
investigation of a mediastinal mass	1/38 (2.6%)
investigation of abnormal shadow in chest X-ray	1/38 (2.6%)
screening for collagen disease	1/38 (2.6%)

The number and percentage of the bronchiole with the distance shorter than 10 mm are summarized in Table 2. The radiation doses are summarized in Table 3.

**Table 2:** Distances between peripheral bronchial ends and the nearest pleural surface in Group5, Group10, and Group10Thin.

	Group5	Group10	Group10Thin
Distance between the bronchial ends and the pleura (mm, mean ± standard error)	10.5 ± 0.07*^†^^‡^	8.9 ± 0.04*^†^^§^	8.5 ± 0.05*^‡^^§^
Number of bronchial ends found within 10mm of the pleura (%)	98/228 (43.0%)	152/228 (66.7%)	160/228 (70.2%)

*Group5 vs. Group10 vs. Group10Thin: p < 0.001 (Friedman test).

^†^ Group5 vs. Group10: p < 0.001.

^‡^ Group5 vs. Group10Thin: p < 0.001.

^§^ Group10 vs. Group10Thin: p < 0.001.

^†‡§^ *p* values using Wilcoxon signed-rank test with Bonferroni correction (tripled original *p* values).

**Table 3:** Radiation doses (mean ± standard error).

total CTDIvol (mGy)	10.9 ± 0.12
total DLP (mGy.cm)	465 ± 6.6
Effective dose (mSv)	6.5 ± 0.092

CTDIvol = Volume computed tomography dose index, DLP = dose length product.

## Discussion

The visible bronchial tube ends were significantly more peripheral in the 1024 matrix groups than in the 512 matrix group. There was also significant difference in visualization between 0.5-mm slice thickness/increment and 0.25-mm slice thickness/increment in the 1024 matrix groups. The bronchiole ends were found within 10 mm of the pleura in many patients. To our knowledge, this is the first study to compare the visualization of the bronchiole end with different axial matrix sizes, as well as different thicknesses and increments in QDCT.

Hata et al. have compared the effect of matrix size on the visualization of lung structures, such as small vessels, bronchi, and nodules, in QDCT and reported that image quality of bronchial tubes was better with 1024 × 1024 and 2048 × 2048 matrix sizes than with the 512 × 512 matrix size, which is consistent with our finding [12]. Tanabe et al. have compared the visualization of the bronchial lumen and bronchial wall thickness between the 512 × 512 matrix and 1024 × 1024 matrix in QDCT and reported much better accuracy with the 1024 × 1024 matrix [13].

It is considered that bronchioles are not found within 10 mm from the pleura on CT in the normal population and that the presence of bronchial tubes within 10 mm from the pleura indicates bronchiectasis [9], and the latest guidelines for bronchiectasis use the same standard. According to the British Thoracic Society Guidelines for Bronchiectasis in Adults, the following CT imaging findings are considered to indicate bronchiectasia: bronchoarterial ratio >1, lack of bronchial tapering, and visibility of airways within 10 mm of the pleural surface or touching the mediastinal pleural surface [10]. However, in the present study, within 10 mm from the pleura, many bronchioles were demonstrated in 98/228 parts in Group 5 and in 152 and 160/228 parts in Group 10 and Group 10Thin, respectively. These findings suggest that visibility of bronchioles within 10 mm from the pleura is not always pathologic, especially in QDCT.

The present study has some limitations. First, the number of patients was small. Second, there was a potential influence of measurement error. Third, all images were assessed only in the axial view.

## Conclusions

In conclusion, the visualization of peripheral bronchioles in QDCT was much better with a 1024 × 1024 axial matrix than with a 512 × 512 matrix, and with a 0.25-mm slice thickness/increment than with a 0.5-mm slice thickness/increment. The identification of bronchial tubes within 10 mm from the pleura on QDCT might not always be pathologic.
